# Transcriptome-Based Selection and Validation of Reference Genes for Gene Expression Analysis of *Alicyclobacillus acidoterrestris* Under Acid Stress

**DOI:** 10.3389/fmicb.2021.731205

**Published:** 2021-08-27

**Authors:** Ning Zhao, Junnan Xu, Lingxia Jiao, Mengzhen Qiu, Jie Zhang, Xinyuan Wei, Mingtao Fan

**Affiliations:** ^1^College of Food Science and Engineering, Northwest A&F University, Xianyang, China; ^2^School of Food Science, Henan Institute of Science and Technology, Xinxiang, China

**Keywords:** *ctsR*, *dnaG*, geNorm, juice spoilage, normalization, RefFinder

## Abstract

Alicyclobacillus *acidoterrestris* is a major concern in fruit juice industry due to its spoilage potential of acidic fruit juice. Quantifying the expression levels of functional genes by real-time quantitative polymerase chain reaction (RT-qPCR) is necessary to elucidate the response mechanisms of *A. acidoterrestris* to acid stress. However, appropriate reference genes (RGs) for data normalization are required to obtain reliable RT-qPCR results. In this study, eight novel candidate RGs were screened based on transcriptome datasets of *A. acidoterrestris* under acid stress. The expression stability of eight new RGs and commonly used RG 16s rRNA was assessed using geNorm, NormFinder, and BestKeeper algorithms. Moreover, the comprehensive analysis using the RefFinder program and the validation using target gene *ctsR* showed that *dnaG* and *dnaN* were the optimal multiple RGs for normalization at pH 4.0; *ytvI*, *dnaG*, and 16s rRNA at pH 3.5; *icd* and *dnaG* at pH 3.0; and *ytv*I, *dnaG*, and *spoVE* at pH 2.5. This study revealed for the first time that *A. acidoterrestris* had different suitable RGs under different acid conditions, with implications for further deciphering the acid response mechanisms of this spoilage-causing bacterium.

## Introduction

Commercial fruit juices have been widely regarded as safe from pathogenic and spoilage-causing microorganisms due to their inherent high acidity, unfavorable for microbial survival, growth, and proliferation (Kakagianni et al., 2018). However, a large-scale spoilage incident of pasteurized apple juice occurred in 1982, in which *Alicyclobacillus acidoterrestris* was identified as the causative agent (Cerny et al., 1984). Thereafter, *A. acidoterrestris* related contamination has been reported in various fruit juices and beverages (Smit et al., 2011; Pornpukdeewattana et al., 2020).

*Alicyclobacillus acidoterrestris* is a Gram-positive, spore-forming, thermo-acidophilic and strictly aerobic bacterium (Smit et al., 2011). The unique heat and acid resistance of *A. acidoterrestris* enable it to survive commercial pasteurization and further grow and multiply in acidic fruit juice to produce distinct antiseptic, disinfectant, or medicinal off-flavors, causing product deterioration. Such incidents of spoilage have caused considerable economic loss and posed a challenge for the microbial stability of fruit juices worldwide (Witthuhn et al., 2012). Hence, it is imperative to develop effective detection and control measures to avoid the contamination of this bacterium without affecting the nutrition and flavor of fruit juice (Pornpukdeewattana et al., 2020). To achieve this goal, the in-depth profile of the molecular regulatory mechanism of *A. acidoterrestris* resistance to heat and acid stress is essential. Specifically, the expression pattern of target genes under stress conditions must be fully elucidated.

The quantification of functional gene expression levels is one of the most important aspects in systematic clarifying gene transcription and regulation (Chen et al., 2020). To date, the reverse transcription quantitative real-time polymerase chain reaction (RT-qPCR) method has been the preferred method to quantify the target gene expression level due to its high accuracy, sensitivity, and reproducibility. It is also commonly used to confirm the result obtained by RNA sequencing (RNA-seq) (Shakeel et al., 2017). However, the RT-qPCR assay relies on the stably expressed reference genes (RGs) to obtain expression data of genes in different spatial-temporal conditions. An inadequate use of RG may lead to biased gene expression profiles and low reproducibility (Huggett et al., 2005; Guenin et al., 2009; Chen et al., 2020). More and more evidence shows that the expression of RGs fluctuates considerably under different conditions, and no single RG is suitable for all gene expression analysis assays. The main factors introducing the instability of RGs expression include different species, cell types, tissues, cell developmental stages, and experimental treatment conditions (Everaert et al., 2011; Park et al., 2015; Peng et al., 2018; de Lázaro and Kostarelos, 2019). In previous studies, genes such as glyceraldehyde-3-phosphate dehydrogenase (GAPDH), β-actin (ACTB), β-tubulin 2 (β-TUB2), ubiquitin C (UBC), and rRNA are often used as RGs because of their functions in basic cellular processes (Fulvio et al., 2020; Gonzalez-Pujana et al., 2020). However, several studies have shown that these traditional RGs have variable expression levels and are not stably expressed in all experimental conditions. Glare et al. (2002) and Everaert et al. (2011) found that the GAPDH and ACTB displayed high expression variability across healthy and diseased mouse tissues. Meanwhile, the 16s rRNA demonstrated large variability in stability for many bacterial organisms depending on the conditions studied (Liu et al., 2013; Peng et al., 2018). Thus, it is better to evaluate the utility of potential RGs under specific experimental conditions.

For *A. acidoterrestris*, all current studies related to stress responses at the molecular level have used only a single RG (16s rRNA) (Jiao et al., 2015; Feng et al., 2019; Zhao et al., 2021). No reports are available about credible RG selection in *A. acidoterrestris*, which limits further disclosure on its stress tolerance mechanisms. Therefore, a systematic research on stably expressed RGs of *A. acidoterrestris* under stress conditions is required. The RNA-seq technique provides not only a method to determine gene expression at the transcriptome level but also a novel approach for RGs prediction, which has been successfully applied in multiple species (Alexander et al., 2012; He et al., 2019; Zhang et al., 2020; Jureckova et al., 2021). Therefore, it was hypothesized that the novel and reliable RGs for investigating stress responses in *A. acidoterrestris* could be predicted using transcriptome data.

**FIGURE 1:**
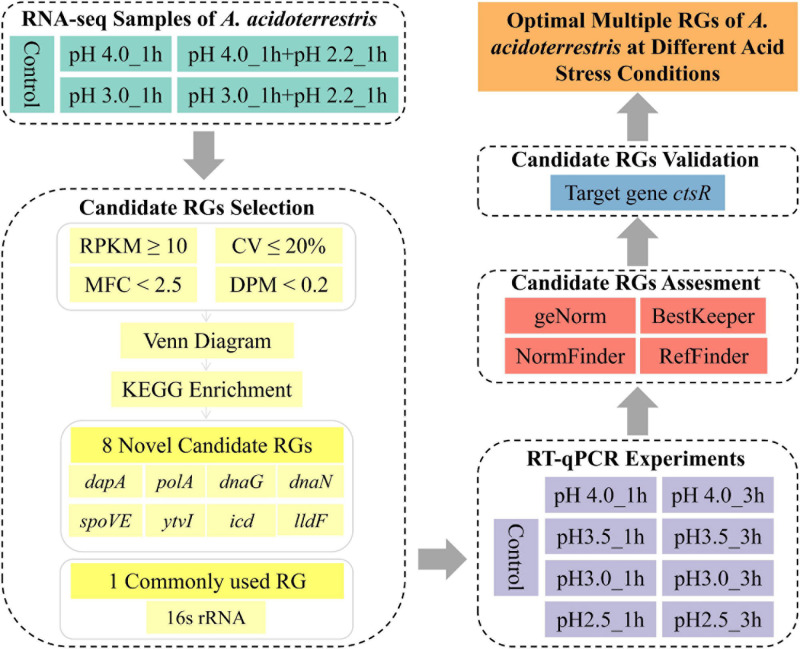
The overall workflow of this study.

In the present study, the transcriptome data of fifteen acid stress-related samples of *A. acidoterrestris* were analyzed. Potential RGs were predicted on a genome-wide scale based on their relatively stable expression levels, of which eight genes (*polA*, *icd*, *dnaG*, *dapA*, *dnaN*, *ytvI*, *spoVE*, and *lldF*) were selected. The commonly used RG (16s rRNA) was selected for comparison. These nine candidate RGs were amplified using RT-qPCR in nine groups of samples with different acid stress treatments. Three different algorithms (geNorm, NormFinder, and BestKeeper) and a comprehensive program (RefFinder) were used to evaluate the stability of each RG. Finally, the reliability of recommended optimal RGs was validated using the target gene (*ctsR*). The overall workflow of this study is shown in Figure 1. The results of this study might serve as potential resources for dissecting the response mechanisms of *A. acidoterrestris* to acid stress.

## Materials and Methods

### Bacterial Strain and Growth Conditions

*A. acidoterrestris* DSM 3922^T^ was purchased from Deutsche Sammlung von Mikroorganismen und Zellkulturen (DSMZ, Braunschweig, Germany). The *A. acidoterrestris* medium (AAM) prepared following the method proposed by Jiao et al. (2015) was used as culture medium. The bacterial stock culture was thawed at room temperature and then streaked onto AAM agar incubating at 45°C for 24 h. Then, a single colony was transferred to 50 mL of AAM broth and incubated at 45°C with shaking at 150 rpm for 12 h. The activated culture was obtained after a consecutive transfer (2%, v/v) under the same conditions.

### Preparation of Acid Stress Samples

Cells grown to the mid-log phase (6 h) were divided into five equal parts, centrifuged, and washed with sterile saline. Four parts of cell pellets were suspended in the same volume of fresh AAM broth with adjusted pH values (pH 3.5, 3.0, and 2.5) or normal AAM broth (pH 4.0), and then incubated at 45°C with shaking at 150 rpm. The pH values were adjusted by addition of 1 mol/L HCl. Samples were collected at 1 h and 3 h after acid stress. The mid-log phase cells without acid stress was used as a control. All treatments were performed in triplicate using independent cultures. The collected cells were washed, and the cell pellets were lyophilized immediately using liquid nitrogen and subsequently subjected to RNA extraction.

### RNA Extraction and cDNA Synthesis

Total RNA was extracted using an RNAiso Kit following the manufacturer’s protocols (TakaRa, Japan). The quality and concentration of RNA samples were determined using a NanoDrop 2000 spectrophotometer (Thermo Scientific, United States), and the integrity was further analyzed in Agilent 2100 (Agilent Technologies, DE). The qualified RNA was reverse transcribed to cDNA using a PrimeScript RT reagent kit with gDNA Eraser (TakaRa, Japan) (He et al., 2018). The reverse transcription assay (20 μL volume) was performed according to the following steps: 37°C for 15 min, 85°C for 5 s, cool down to 4°C. cDNA was stored at −20°C until RT-qPCR analyses.

### Candidate RGs Selection

Transcriptome sequencing of *A. acidoterrestris* under acid stress was performed by RNA-seq on an Illumina HiSeqTM2500 platform (unpublished data, Figure 1). The values of reads per kilobase of transcript per million mapped reads (RPKM) were calculated using Cufflinks after quality control and filtration of low-quality raw sequence data (Park et al., 2018). The coefficient of variation (CV,%) and maximum fold change (MFC) were calculated using Microsoft Excel based on the RPKM value of every gene in each sample. The CV is defined as the ratio of the standard deviation (SD) to the mean of the FPKM of all samples for one gene, while the MFC is defined as the fold change between the largest and smallest RPKM values within all samples (Zhang et al., 2020). Meanwhile, the dispersion measure (DPM) was calculated on the pattern gene finder (PaGeFinder) website^1^ (Pan et al., 2012). Probability density curves of FPKM, CV, DPM, and MFC of transcripts with credible function annotation and high expression levels (RPKM ≥ 10) were fitted and drawn using the Origin software. Venn diagram analysis and Kyoto Encyclopedia of Genes and Genomes (KEGG) enrichment was performed using the OmicShare online platform tools^2^.

### Primers Design and Amplification Efficiency Analysis

Specific primer pairs for candidate RGs and target gene were designed using Primer 5 and are listed in Table 1. Primers were synthesized by AuGCT DNA-SYN Biotechnology Company (Beijing, China), and further purified with polyacrylamide gel electrophoresis (PAGE) method. Amplification efficiencies (*E*) and correlation coefficients (*R*^2^ values) of genes were generated using slopes of the standard curves obtained from tenfold diluted cDNA series. The *E*-value was calculated using the formula: *E* (%) = [10^(–1/slope)^ − 1] × 100.

**TABLE 1:** Gene descriptions and primer sequences used for RT-qPCR.

**Gene ID**	**Gene description**	**Gene symbol**	**Primer sequence (5′ → 3′)**	**PCR product length (bp)**
N007_20375	DNA primase	*dnaG*	F: GGATTTTCGCTTAGGGTTTG	239
			R: GCGAGTTCAGGTATTTAGGT	
N007_04785	DNA polymerase III subunit β	*dnaN*	F: GACAAGTGAGCGAGTGGTTC	248
			R: GAAACAGCGCGTATGGAACT	
N007_01100	Stage V sporulation protein E	*spoVE*	F: CCGCAGACCGATTTTATTTT	141
			R: TAAAGACCCAAACTCATCCG	
N007_01250	Sporulation integral membrane protein	*ytvI*	F: AAGTGTTCTATGGGCAGATG	110
			R: AAGAGACCTGACAAAACTCC	
N007_05875	DNA polymerase I	*polA*	F: GTCGGGTTCCCTTGTAGT	138
			R: CAAATGCGGTGTATGGTT	
N007_09410	Lactate utilization protein	*lldF*	F: CCATGATTACCGGCCCAAAG	158
			R: CGGGCAGACGTTTAGACAAG	
N007_05885	Isocitrate dehydrogenase	*icd*	F: TCACCTTGTCTAAATTCGCA	202
			R: CAGCAAATTCTTACACGTCC	
N007_06870	4-Hydroxy-tetrahydrodipicolinate synthase	*dapA*	F: GGCTGACCAATCAACTCCA	286
			R: TTACTCAAATCTCCCACATCG	
N007_09120	16S Ribosomal RNA	16s rRNA	F: GCATGAAGCCGGAATTGCTA	126
			R: AACGGTTACCTCACCGACTT	
N007_07600	Transcriptional regulator	*ctsR*	F: GCATACTCTGTTGACCTTGTTG	140
			R: CTCTCCGCAGCATATTCTCC	

### RT-qPCR Analysis

The assays were carried out in a 25-μL reaction containing 12.5 μL of TB Green *Premix Ex* Taq (Tli RNaseH Plus) (TakaRa, Japan), 1.0 μL each of the forward and reverse primers (0.4 μM), 2 μL of template cDNA (<100 ng), and 8.5 μL of nuclease-free water. Quantitative amplification was performed in a CFX-96 thermal cycler (Bio-Rad Laboratories, United States) as follows: 1 cycle at 95°C for 30 s, 40 cycles at 95°C for 5 s, and 60°C for 30 s. To confirm product specificity, a melting curve analysis between 60 and 95°C was performed after each amplification. No-template and no-RT controls for each primer pair were included. Each RT-qPCR was performed in triplicate, and each experiment was independently repeated three times.

### Determination of the Expression Stability of RGs

The expression level of candidate RGs was evaluated according to the quantification cycle (*C*q) value. Three statistical algorithms, geNorm (Vandesompele et al., 2002), BestKeeper (Pfaffl et al., 2004), and NormFinder (Andersen et al., 2004) were used to determine the expression stability of candidate RGs. Raw *C*t values were directly applied for BestKeeper analysis, but the raw data were converted into relative quantities using the 2^–Δ^*^C^*^t^ method (Δ*C*t = eachCt − minimumCt) for the other two analyses. Finally, the online tool RefFinder^3^ was used to confirm whether the three algorithms worked properly and to comprehensively rank RGs from experimental data (Xie et al., 2012).

### Validation of the Selected RGs

To validate the selected RGs, the expression level of one target gene (*ctsR*) was detected with the same cDNA samples used to select RGs. The RT-qPCR conditions were the same as previously described in Section “RT-qPCR Analysis.” The relative expression level of the *ctsR* gene was calculated using the 2^–ΔΔ^*^C^*^t^ method (Livak and Schmittgen, 2001). Four RG strategies were used for data normalization, including the optimal multiple RGs from each treatment, the optimal multiple RGs from all samples, the least stable RG from each treatment, and the commonly used RG (16s rRNA) (Peng et al., 2018). The Δ*C*t data was used to calculate statistical differences using the one-way analysis of variance (ANOVA) employing Duncan’s test at *p* < 0.05. Statistical analysis was performed using SPSS version 19.0 (IBM, United States). Three replicates of four independent experiments were performed.

### Compliance With the MIQE Guidelines

The RT-qPCR analysis in this work was performed in strict compliance with the minimum information for publication of quantitative real-time PCR experiments (MIQE) guidelines (Bustin et al., 2009) to promote transparency and ensure the reliability and integrity of the results. All experimental procedures were carried out in the investigators’ laboratory, with the exception of the RNA quality assessment, which was performed with Agilent 2100 at the College of Agronomy of Northwest A&F University. The MIQE checklist is detailed in Supplementary Table 6.

## Results

### Selection of Candidate RGs Based on RNA-Seq Data

**FIGURE 2:**
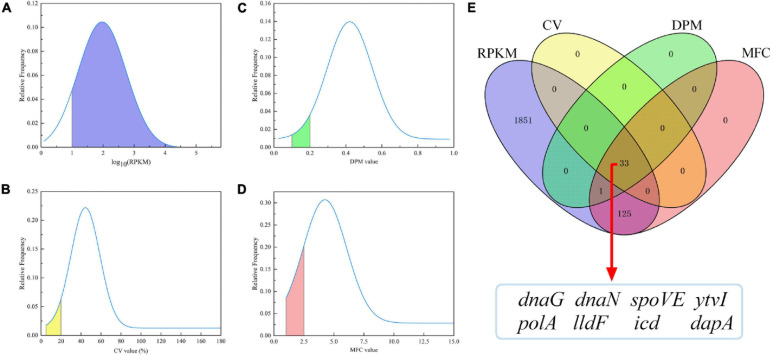
Probability density curve of RPKM, CV, DPM, and MFC values of 2751 genes **(A–D)**, and the overlap genes obtained by the Venn diagram analysis **(E)**. RPKM, reads per kilobase of transcript per million mapped reads; CV, coefficient of variation; DPM, dispersion measure; MFC, maximum fold change.

RPKM values of all transcripts in each *A. acidoterrestris* sample were obtained from transcriptome datasets. 2,751 unigenes were first chosen for further RG_S_ selection after removing transcripts without a credible functional annotations or with low expression levels (RPKM = 0). Then, the probability density curve of all 2,751 genes was evaluated using four indicators including RPKM, CV, MFC, and DPM values (Figures 2A–D). Potential RGs were relatively highly expressed genes (Brown et al., 2018). A total of 2010 genes had RPKM values ≥ 10, accounting for 73.06% of all 2,751 genes (blue area, Figure 2A). Meanwhile, the most promising RGs had low expression variability, namely, the lowest CV, MFC, and DPM values (Gao et al., 2018; Zhang et al., 2020). As shown in Figure 2B, 33 genes had a CV value ≤ 20% (1.20% of 2751 genes, yellow area). A more stringent DPM (<0.2) than the default parameter (<0.3) was used. A total 34 genes had the DPM values < 0.2 (1.24% of 2,751 genes, green area) (Figure 2C). The MFC reflects the range of extreme values, and there were 159 genes with MFC < 2.5 (5.78% of 2,751 genes, pink area, Figure 2D).

A Venn diagram analysis was further performed (Figure 2E), in which blue, yellow, green, and pink corresponded to those shown in Figures 2A–D, respectively. A total of 33 genes simultaneously met the aforementioned requirements of four parameters, which were significantly enriched in four KEGG pathways (*q* < 0.05) (Supplementary Table 1) and were considered as candidate RGs. Of these, eight genes (*dapA*, *spoVE*, *ytvI*, *polA*, *icd*, *dnaG*, *lldF*, and *dnaN*) with a relatively lower CV value were selected. The summarized information of eight potential RGs based on transcriptome data and the raw RPKM values are shown in Table 2 and Supplementary Table 2, respectively. In addition, the commonly used RG (16s rRNA) for exploring target gene expression in *A. acidoterrestris* was included. In total, nine candidate RGs were selected for further assessment.

**TABLE 2:** The summarized information of candidate reference genes in *Alicyclobacillus acidoterrestris* based on the transcriptome data.

**Genes**	**Mean_RPKM**	**CV(%)**	**Ranking order^α^**	**MFC**	**DPM**
*dapA*	220.37	12.18	2	1.56	0.12
*spoVE*	707.98	12.25	3	2.12	0.12
*ytvI*	277.07	12.83	6	1.62	0.13
*polA*	38.01	14.82	8	1.79	0.15
*icd*	58.74	14.98	9	1.58	0.15
*dnaG*	206.91	16.54	16	1.72	0.16
*lldF*	2341.95	18.71	27	1.88	0.18
*dnaN*	238.67	19.23	28	1.77	0.19

*RPKM, reads per kilobase of transcript per million mapped reads; CV(%), coefficient of variation; MFC, maximum fold change, highest/lowest FPKM value of one gene within all acid-stressed samples; DPM, dispersion measure, determined by PaGeFinder method; ^α^ranking order in all genes based on the CV value.*

### Determination of Primer Amplification Specificity and Efficiency

**FIGURE 3:**
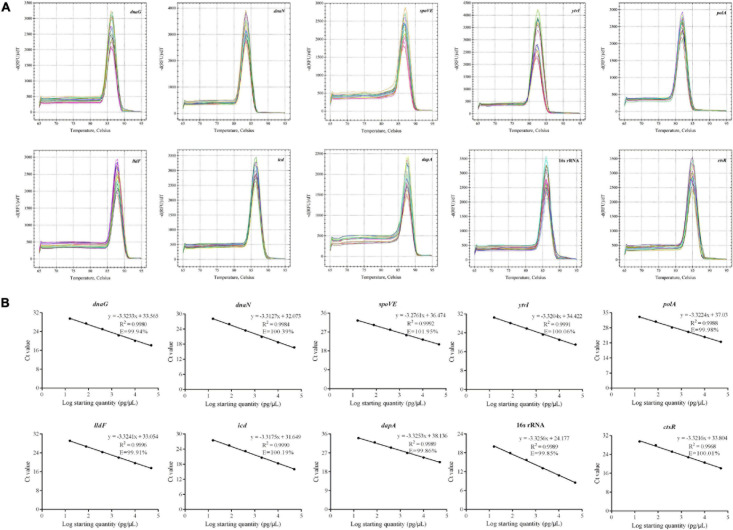
Confirmation of the primer amplification specificity and efficiency of candidate reference genes and target gene. **(A)** Melting curve analysis; **(B)** standard curves showing the correlation between the copies and the quantification cycle (*C*q) values.

As shown in Figure 3A, the melting curves observed from PCR amplification products for each gene had a single distinct sharp peak, indicating the absence of non-specific amplicons or primer dimers. The amplification efficiency varied from 99.85% (16s rRNA) to 101.95% (*spoVE*), and the linear correlation coefficients (*R*^2^) were from 0.9968 (*ctsR*) to 0.9996 (*lldF*) (Figure 3B). Therefore, each pair of primers had good amplification specificity and efficiency, and can be used in RT-qPCR analysis.

### Expression Profiles of Candidate RGs

**FIGURE 4:**
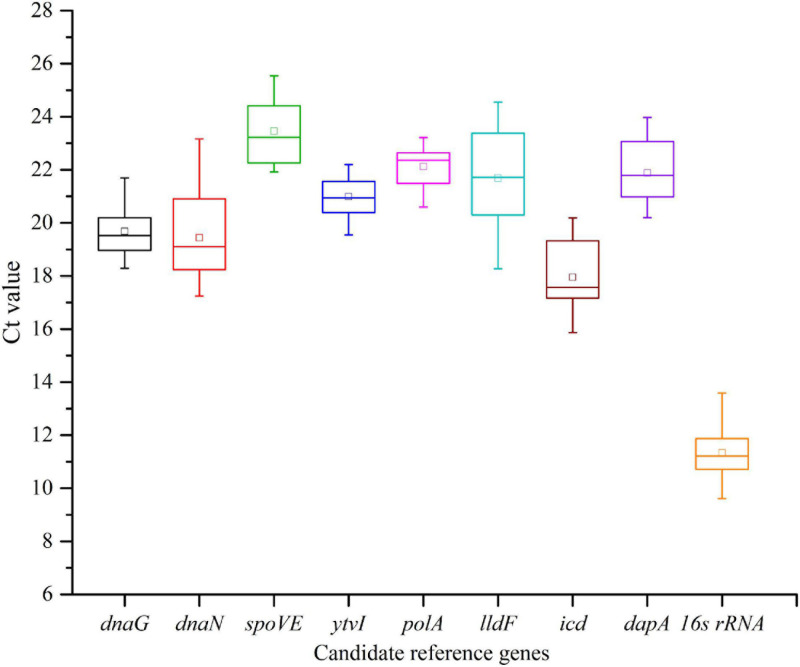
Expression levels of nine candidate reference genes across all samples in *Alicyclobacillus acidoterrestris*. The outer box is determined from 25th to 75th percentiles, and the inner box represents the mean value. The line across the box is the median. The whiskers represent the minimum and maximum values.

The cycle threshold (*C*t) values were detected across all samples to determine the transcriptional differences among nine candidate RGs. Globally, the tested RGs featured a relatively wide expression variation, with the *C*t values ranging from 9.61 to 25.54 (Figure 4 and Supplementary Table 3). The gene with the highest expression level was 16s rRNA, with the lowest mean *C*t value (11.34), and the lowest was *spoVE* with the highest mean *C*t value (23.46). The narrower the SD range of a gene was, the higher its expression stability in different samples was. The top three RGs with low SDs were *polA* (0.734), *ytvI* (0.756), and *dnaG* (0.964), while the three most variably expressed genes were *icd* (1.299), *dnaN* (1.594), and *lldF* (2.011).

### Expression Stability Analysis of Candidate RGs

#### geNorm Analysis

**FIGURE 5:**
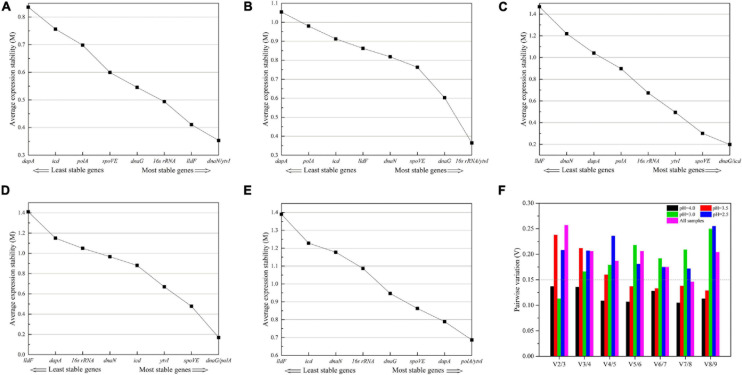
Average expression stability **(A–E)** and optimal number of reference genes **(F)** calculated using geNorm program. **(A–D)** *A. acidoterrestris* cells treated at pH 4.0, 3.5, 3.0, and 2.5, respectively. **(E)** All samples. **(F)** The optimal number of reference genes was calculated based on the pairwise variation (*V*_*n*_/*V*_*n* + 1_) values.

The expression stability of nine candidate RGs was ranked by the *M*-values calculated using geNorm analysis. As shown in Figures 5A–E, *M*-values of nine RGs in each treatment were lower than the default threshold of 1.5, meaning they were stably expressed. Meanwhile, the lower *M*-value suggested a higher expression stability (Vandesompele et al., 2002). For cells cultivated in normal AAM (pH 4.0), the two most stable genes were *dnaN* and *ytvI* with the lowest *M*-value (Figure 5A). Under acid stress at pH 3.5, 16s rRNA and *ytvI* were the two most stable genes (Figure 5B). At pH 3.0, the two most stable genes were *dnaG* and *icd* (Figure 5C). The two most stable genes at pH 2.5 were *dnaG* and *polA* (Figure 5D). The most unstable gene was *dapA* at pH 4.0 and 3.5, and *lldF* at pH 3.0 and 2.5 (Figures 5A–D). Considering all samples, *polA* and *ytvI* were the two most stable genes, and the most unstable gene was *lldF* (Figure 5E).

Meanwhile, the geNorm algorithm determines the optimal number of RGs by stepwise calculation of pairwise variation (*V*_*n*__/__*n*__+__1_) between the two sequential normalization factors NF_*n*_ and NF_*n*__+__1_. When *V*_*n*__/__*n*__+__1_ is less than 0.15, the minimum value of *n* was the optimal number of RG needed (Vandesompele et al., 2002). As shown in Figure 5F, the *V*_2__/__3_ values for pH 4.0 and 3.0 treatments were both lower than the cutoff value of 0.15, indicating that using two RGs was sufficient to normalize qRT-PCR derived gene expression data under these two conditions. However, the *V*_5__/__6_ value was lower than 0.15 at pH 3.5, and the lowest *V*_*n*__/__*n*__+__1_ value was always above 0.15 at pH 2.5. The help center of geNorm software indicated that a cutoff value of 0.15 was not a strict value and suggested the use of three best RGs as a valid normalization strategy. And the threshold of *V*_*n*__/__*n*__+__1_ should be dependent on specific data (Vandesompele et al., 2002; Wan et al., 2010; Fulvio et al., 2020). Considering the results and practical feasibility, three RGs were appropriate for gene expression normalization for *A. acidoterrestris* under pH 3.5 and 2.5 stress conditions.

#### NormFinder Analysis

NormFinder determines the expression stability values of candidate RGs by combining intra- and intertreatment expression variations. Low expression stability values indicated that genes had stable expression (Andersen et al., 2004). As shown in Table 3, *dnaG* was the most stable gene at pH 4.0 and 3.0, while *ytvI* was the most stable gene at pH 3.5, pH 2.5, and in the combined analysis of all samples. At pH 4.0, 3.5, and 3.0, the top three genes analyzed by NormFinder analysis included the two most stable genes obtained from the aforementioned geNorm algorithm. However, at pH 2.5, the two most stable genes from geNorm analysis, *dnaG* and *icd*, ranked sixth and seventh, respectively, in NormFinder. However, the least stable genes identified by both geNorm and NormFinder were the same in different acid-stressed samples.

**TABLE 3:** Expression stability of candidate reference genes in *A. acidoterrestris* assessed by NormFinder program.

**Overall rank**	**pH = 4.0**		**pH = 3.5**		**pH = 3.0**		**pH = 2.5**		**All samples**	
	**Gene**	**SV**	**Gene**	**SV**	**Gene**	**SV**	**Gene**	**SV**	**Gene**	**SV**
1	*dnaG*	0.149	*ytvI*	0.113	*dnaG*	0.069	*ytvI*	0.206	*ytvI*	0.438
2	*dnaN*	0.247	*dnaG*	0.187	*icd*	0.069	*icd*	0.267	16s rRNA	0.458
3	*ytvI*	0.325	16s rRNA	0.451	*spoVE*	0.193	*spoVE*	0.332	*dnaG*	0.623
4	*lldF*	0.363	*icd*	0.459	*ytvI*	0.256	*dnaN*	0.421	*icd*	0.635
5	16s rRNA	0.431	*spoVE*	0.504	16s rRNA	0.477	16s rRNA	0.692	*polA*	0.641
6	*polA*	0.462	*dnaN*	0.643	*polA*	0.983	*dnaG*	0.778	*dnaN*	0.655
7	*icd*	0.477	*polA*	0.671	*dnaN*	1.011	*polA*	0.805	*spoVE*	0.675
8	*spoVE*	0.552	*lldF*	0.728	*dapA*	1.156	*dapA*	1.078	*dapA*	0.759
9	*dapA*	0.692	*dapA*	0.786	*lldF*	1.539	*lldF*	1.577	*lldF*	1.253

*SV, stability value.*

#### BestKeeper Analysis

**FIGURE 6:**
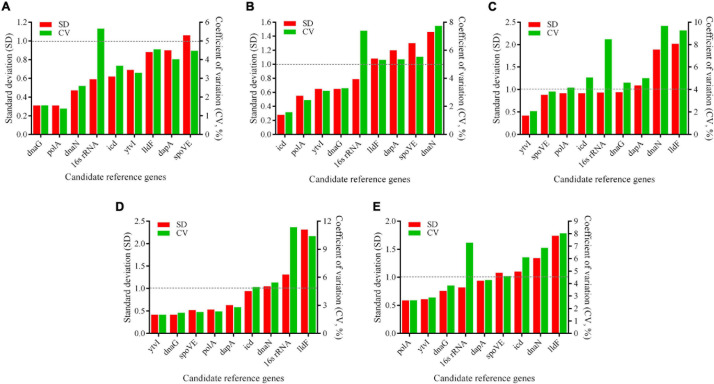
Expression stability of candidate reference genes in *A. acidoterrestris* assessed by BestKeeper program. **(A–D)** *A. acidoterrestris* cells treated at pH 4.0, 3.5, 3.0, and 2.5, respectively. **(E)** All samples. SD, standard deviation; CV, coefficient of variation.

BestKeeper algorithm assesses the expression stability of RGs based on the SD and CV values, with the lower SD value, the more stable RG expression is. Genes with an SD value greater than one were considered unsuitable as RGs (Pfaffl et al., 2004). As shown in Figure 6, SD values of the most stable candidate RGs identified using geNorm and NormFinder were all less than one, further confirming that these genes were stably expressed under different conditions. However, a slight difference was observed between the results of BestKeeper analysis and the findings obtained by aforementioned two methods. According to the BestKeeper algorithm, *dnaG*, *polA*, and *dnaN* were regarded as the three most stably expressed genes at pH 4.0 (Figure 6A), while *polA* ranked sixth in both geNorm and NormFinder analysis. At pH 3.5, *icd*, *polA*, and *ytvI* were the most stable genes (Figure 6B), while *icd* ranked sixth using geNorm, and *polA* ranked seventh using the aforementioned two methods. *ytvI*, *spoVE*, and *polA* were the most stable genes at pH 3.0 (Figure 6C), while *polA* ranked fifth and sixth in geNorm and NormFinder, respectively. At pH 2.5, the most stably expressed genes were *ytvI*, *dnaG*, and *spoVE* (Figure 6D), while *dnaG* ranked fifth using NormFinder.

#### A Comprehensive Analysis

**FIGURE 7:**
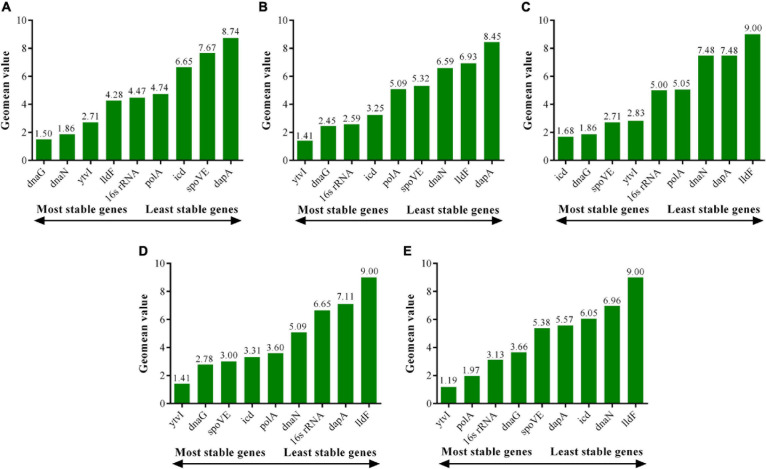
Expression stability of candidate reference genes in *A. acidoterrestris* assessed by RefFinder program. **(A–D)** *A. acidoterrestris* cells treated at pH 4.0, 3.5, 3.0, and 2.5, respectively. **(E)** All samples.

The comprehensive ranking of the expression stability of candidate RGs was obtained by the RefFinder algorithm, which integrated the results of the aforementioned three standard analysis algorithms (Xie et al., 2012). At pH 4.0, the top three most stable RGs were *dnaG*, *dnaN*, and *ytvI*. At pH 3.5, *ytvI*, *dnaG*, and 16s rRNA were the most stable RGs analyzed. At pH 3.0, *icd*, *dnaG*, and *spoVE* were the most stably expressed RGs. At pH 2.5, *ytvI*, *dnaG*, and *spoVE* were the most stable RGs (Figure 7 and Supplementary Table 4). Interestingly, the first three genes with the most stable expression under the four acid stress treatments all included *dnaG*, as shown in Venn diagram analysis (Supplementary Figure 1), indicating that *dnaG* could be used as a stable RG at pH ranging from 4 to 2.5. Except for pH 3.0, *ytvI* was one of the top three stable genes in the other three acid-stressed cells. Meanwhile, *spoVE* was confirmed in the top three stable genes in cells treated with stronger acid stress (pH 3.0 and 2.0).

Combining the optimal number of RGs for normalization obtained using geNorm, *dnaG* and *dnaN* were considered as optimal multiple RGs at pH 4.0, while *dnaG* and *icd* were optimal RGs at pH 3.0. However, three RGs were selected for normalization under the other two acid stress conditions. *ytvI*, *dnaG*, and 16s rRNA were used as ideal multiple RGs for normalization at pH 3.5, and *ytv*I, *dnaG*, and *spoVE* at pH 2.5.

### Validation of the Recommended RGs

**FIGURE 8:**
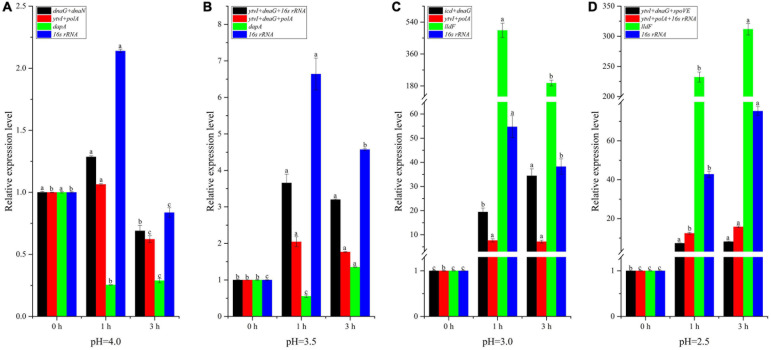
Relative expression level of the *ctsR* gene normalized by the different reference genes (RGs) in *A. acidoterrestris* cells under different acid stress conditions. **(A)** pH = 4.0, **(B)** pH = 3.5, **(C)** pH = 3.0, **(D)** pH = 2.5. Different letters above each column for the same RG normalization strategy indicate significant differences at a significant level of 0.05.

To validate the selected candidate RGs, the expression profile of target gene *ctsR* involved in bacterial stress responses was detected (Figure 8 and Supplementary Table 5). Besides the optimal multiple RGs selected from each treatment, the least stable gene in each treatment (*dapA* at pH 4.0 and 3.5, *lldF* at pH 3.0 and 2.5) and the optimal multiple RGs selected from all samples (*ytvI* and *polA* at pH 4.0 and 3.0; *ytvI*, *dnaG*, and *polA* at pH 3.5; and *ytvI*, *polA*, and 16s rRNA at pH 2.5) were also used to normalize the expression level of *ctsR*. As shown in Figure 8A, the *ctsR* was upregulated at pH 4.0 for 1 h but downregulated for 3 h when normalized by optimal multiple RGs. After acid stress at pH 3.5 for both 1 h and 3 h, the expression level of *ctsR* increased when normalized by optimal multiple RGs (Figure 8B). However, a decreased pattern was discovered at pH 4.0 and pH 3.5 for 1 h when normalized with the least stable genes. Overall, the expression of *ctsR* was underestimated when normalized by the least stable gene at pH 4.0 and 3.5 compared with that normalized by optimal multiple RGs. Using these three normalization methods, the expression level of *ctsR* increased after 1 h and 3 h at both pH 3.0 and 2.5 (Figures 8C,D). However, when normalized using the least stable genes, the expression level exhibited an obvious overestimation with a sharp increasing pattern.

## Discussion

Gene expression analysis is a primary method to reveal the response mechanism of microorganisms to different stress conditions, and several techniques have been developed to investigate gene expression at the transcriptional level. RT-qPCR is one of the most utilized methods because it can quantify the transcript abundance of target genes in an accurate and reproducible manner (Bernáldez et al., 2017). However, correct normalization using appropriate RGs is a prerequisite for the reliable assessment of gene expression in RT-qPCR analysis (Guenin et al., 2009). In this study, RGs suitable for quantifying the expression level of genes involved in acid responses of *A. acidoterrestris*, a spoilage-causing bacterium in acidic fruit juice, were selected and evaluated. The acid stress conditions applied (pH 4.0, 3.5, 3.0, and 2.5) were selected based on the growth profiles of *A. acidoterrestris* at different pH values (Zhao et al., 2021) and the actual acidity of fruit juices (Smit et al., 2011).

A total of eight candidate novel RGs were selected from the transcriptome data of *A. acidoterrestris* using the criteria including high expression levels (RPKM ≥ 10), and a low variability with CV ≤ 20%, MFC < 2.5, and DPM < 0.2 (Figure 2). Multiple studies have shown that transcriptomic data is a reliable source for exploring suitable RGs under specific experimental conditions, and similar criteria have been used for RGs screening in other species (Gao et al., 2018; Zhang et al., 2020; Xing et al., 2021). For *A. acidoterrestris*, all current studies on the functional gene expression used 16s rRNA as RG to normalize RT-qPCR data, but the stability of 16s rRNA has not been evaluated under specific conditions (Jiao et al., 2015; Feng et al., 2019; Zhao et al., 2021). Therefore, 16s rRNA was also selected as a candidate RG to evaluate its expression stability under acid stress.

For all candidate RGs, the Ct values of RT-qPCR were systematically analyzed by geNorm, NormFinder, and BestKeeper methods to obtain the most stably expressed RGs and their best combination for *A. acidoterrestris* under acid stress. A slightly different pattern of the expression stability ranking of candidate RGs was discovered among three program analysis results for the same group of acid-stressed cells (Supplementary Table 4), which was also reported in previous reports (Peng et al., 2018). The variance in results might be due to the differences in algorithms and analytical procedures of these three different methods (Thellin et al., 2009; Noti et al., 2015). Moreover, the function and regulation pattern of genes might also lead to the inaccurate assessment of expression stability ranking. The coregulated genes with similar expression profiles can receive preferential stability ranking from the geNorm algorithm, while NormFinder and BestKeeper are less sensitive to coregulation (Andersen et al., 2004; Pfaffl et al., 2004). To counteract potential bias and avoid contradictory results caused by the use of single methods, the RefFinder program was further used for the comprehensive ranking of candidate RGs. The results showed that the expression stability of candidate RGs was different under different acid conditions (Figure 7 and Supplementary Table 4), implying the necessity for RGs validation under specific experimental treatments. At pH 4.0, *dnaG* was identified as the most stable gene, followed by *dnaN* and *ytvI*. Similarly, *dnaG* and *ytvI* were also positioned in the top three of expression stability ranking at pH 3.5, and *ytvI* was the most stable gene. At pH 3.0 and 2.5, the most stable genes were *icd* and *ytvI*, respectively, followed by *dnaG* and *spoVE* under both conditions. It was interesting to discover that the expression stability of *dnaG* was ranked in the top three under the four acid stress conditions applied. Hence, *dnaG* was a universal RG stably expressed in *A. acidoterrestris* under acid stress. The *dnaG* encodes DNA primase, which primes DNA synthesis by incorporating ribonucleotides complementary to the DNA template to generate an RNA primer. Another point of concern was *spoVE*, encoding the stage V sporulation protein E, which had low expression stability under normal growth (pH 4.0) and low acid stress intensity (pH 3.5). However, at pH 3.0 and 2.5, its expression stability was relatively high, ranking the third, which might be associated with the sporulation of *A. acidoterrestris* under more severe stress conditions. Our previous study found that the dormant spores of *A. acidoterrestris* were produced under acid stress with pH less than 3.5 (Zhao et al., 2021).

Besides evaluating the expression stability of candidate RGs and obtaining the most stable RGs, determining the optimal number of RGs is an important aspect for RT-qPCR assay. As reported in the MIQE guidelines, normalization based on a single RG is not acceptable, and the optimal number of RGs must be experimentally determined (Bustin et al., 2009). Moreover, many studies indicated that the normalization with multiple RGs was necessary for the accurate assessment of gene expression under specific conditions (Noti et al., 2015; Zhang et al., 2020). The geNorm algorithm is usually used to determine the optimal number of RGs based on the pairwise variation (*V*_*n*__/__*n*__+__1_) (Vandesompele et al., 2002). At pH 4.0 and 3.0, the *V*_2__/__3_ value was less than the cutoff value of 1.5, indicating that two RGs would be sufficient for normalizing gene expression (*dnaG* and *dnaN*, and *icd* and *dnaG*, respectively). However, the *V*_5__/__6_ value was less than 0.15 at pH 3.5, indicating that at least five RGs should be included for evaluating gene expression. In addition, the lowest *V*_*n*__/__*n*__+__1_ value was always greater than 0.15 at pH 2.5. Notably, the information about the optimal number of RGs was provided only by geNorm software, which was therefore limited to the ranking obtained by this algorithm and did not consider the results produced by others. Many studies showed that 0.15 should not be considered as an absolute threshold, and the number of RGs had to be a tradeoff between accuracy and functionality (Vandesompele et al., 2002; Wan et al., 2010; Fulvio et al., 2020). Meanwhile, the additional cost due to excessive RGs could limit the number of samples that could be tested (Metcalf et al., 2010). Hence, considering the analysis results and practical feasibility, three RGs would be appropriate for gene expression normalization in *A. acidoterrestris* at pH 3.5 (*ytvI*, *dnaG*, and 16s rRNA) and 2.5 (*ytvI*, *dnaG*, and *spoVE*).

Multiple recent studies revealed that the expression levels of some traditional RGs varied considerably under certain conditions and were not as stable as previously thought (Vandecasteele et al., 2002; Desroche et al., 2005). Similarly, the traditional RG (16s rRNA) for *A. acidoterrestris* was not stably expressed under all the four acid stress conditions used in this study. At pH 4.0, 3.0, and 2.5, 16s rRNA was not a stably expressed RG, with the stability ranking below the fifth out of nine candidate RGs. However, at pH 3.5 and four groups of samples analyzed together, the expression stability of 16s rRNA ranked third and it could be used as a stable RG. Hence, it was too absolute to think that 16s rRNA was completely unsuitable for use as a RG, but caution should be taken in assuming that 16s rRNA is stably expressed under all conditions, and its applicability should be evaluated under each specific experimental condition. The unstable expression of 16s rRNA may be caused by its high transcription level (Desroche et al., 2005), as 16s rRNA had the lowest mean *C*t value (Figure 4). Therefore, the templates for 16s rRNA amplification should be diluted more times than that for other genes in RT-qPCR to neutralize the effects of its high abundance.

In order to validate the selected RGs, three RGs strategies were employed to normalize the expression level of target gene *ctsR*, encoding a transcriptional regulator of the bacterial stress responses (Periago et al., 2002). The expression pattern of *ctsR* was obviously influenced by the RG strategy (Figure 8). When normalized by the optimal RGs, except for cells treated at pH 4.0 for 3 h, the transcription level of *ctsR* increased in the other seven samples compared with control. Similar expression trends of *ctsR* were observed in our previous study in which all acid-stressed cells were analyzed together and 16s rRNA was used as RG (Zhao et al., 2021). Importantly, the expression of 16s rRNA was relatively stable and ranked third when all acid-stressed cells were analyzed together (Figure 7). During normalization with the least stable gene, the expression level of *ctsR* was underestimated at pH 4.0 and 3.5, but overestimated at pH 3.0 and 2.5. Therefore, using reliable RGs was a prerequisite for accurate RT-qPCR data analysis of *A. acidoterrestris* under different acid stress conditions.

## Conclusion

This study illustrated the potential of transcriptomic datasets of *A. acidoterrestris* for identifying novel candidate RGs for RT-qPCR assay and the importance of experimental validation of gene expression stability before selecting suitable RGs under different conditions. Eight candidate genes (*dapA*, *spoVE*, *ytvI*, *polA*, *icd*, *dnaG*, *lldF*, and *dnaN*) with high expression level and low expression variance were screened using transcriptomic datasets generated in *A. acidoterrestris* under acid stress. Three statistical algorithms (geNorm, NormFinder, and BestKeeper), a comprehensive program (RefFinder) analysis, and validation using target gene *ctsR* indicated that *dnaG* and *dnaN* at pH 4.0, *ytvI*, *dnaG*, and 16s rRNA at pH 3.5, *icd* and *dnaG* at pH 3.0, and *ytv*I, *dnaG*, and *spoVE* at pH 2.5 stably expressed and were selected as ideal RGs. Significantly, 16s rRNA of *A. acidoterrestris* was not stably expressed under all acid conditions (pH 4.0–2.5), while *dnaG* could be used as a suitable RG to normalize gene expression under these treatments. This study was the first to systematically analyze the RGs for RT-qPCR in *A. acidoterrestris* under stress conditions. The results will contribute to future investigation of functional genes related to acid responses of *A. acidoterrestris* and facilitate RG selection of other *Alicyclobacillus* strains under different experimental conditions.

## Data Availability Statement

The datasets generated for this study can be found in online repositories. The names of the repository/repositories and accession number(s) can be found below: SRA, PRJNA742261 and PRJNA721075.

## Author Contributions

NZ: conceptualization, methodology, investigation, visualization, and writing-original draft. JX: methodology, validation, and visualization. LJ: conceptualization and validation. MQ: investigation. JZ: investigation and formal analysis. XW: writing-review and editing. MF: conceptualization, supervision, funding, and writing-review and editing. All authors contributed to the article and approved the submitted version.

## Conflict of Interest

The authors declare that the research was conducted in the absence of any commercial or financial relationships that could be construed as a potential conflict of interest.

## Publisher’s Note

All claims expressed in this article are solely those of the authors and do not necessarily represent those of their affiliated organizations, or those of the publisher, the editors and the reviewers. Any product that may be evaluated in this article, or claim that may be made by its manufacturer, is not guaranteed or endorsed by the publisher.

## Footnotes

1
http://bioinf.xmu.edu.cn/PaGeFinder/index.jsp

2
https://www.omicshare.com/tools/

3
https://www.heartcure.com.au/reffinder/
