# Supplementary material for: Transcriptome-Based Selection and Validation of Reference Genes for Gene Expression Analysis of Alicyclobacillus acidoterrestris Under Acid Stress
Source: Front Microbiol. 2021 Aug 27;12:731205. doi: 10.3389/fmicb.2021.731205 (PMC8430261; doi:10.3389/fmicb.2021.731205)
Supplement: Supplementary file 1 [file Data_Sheet_1.PDF]

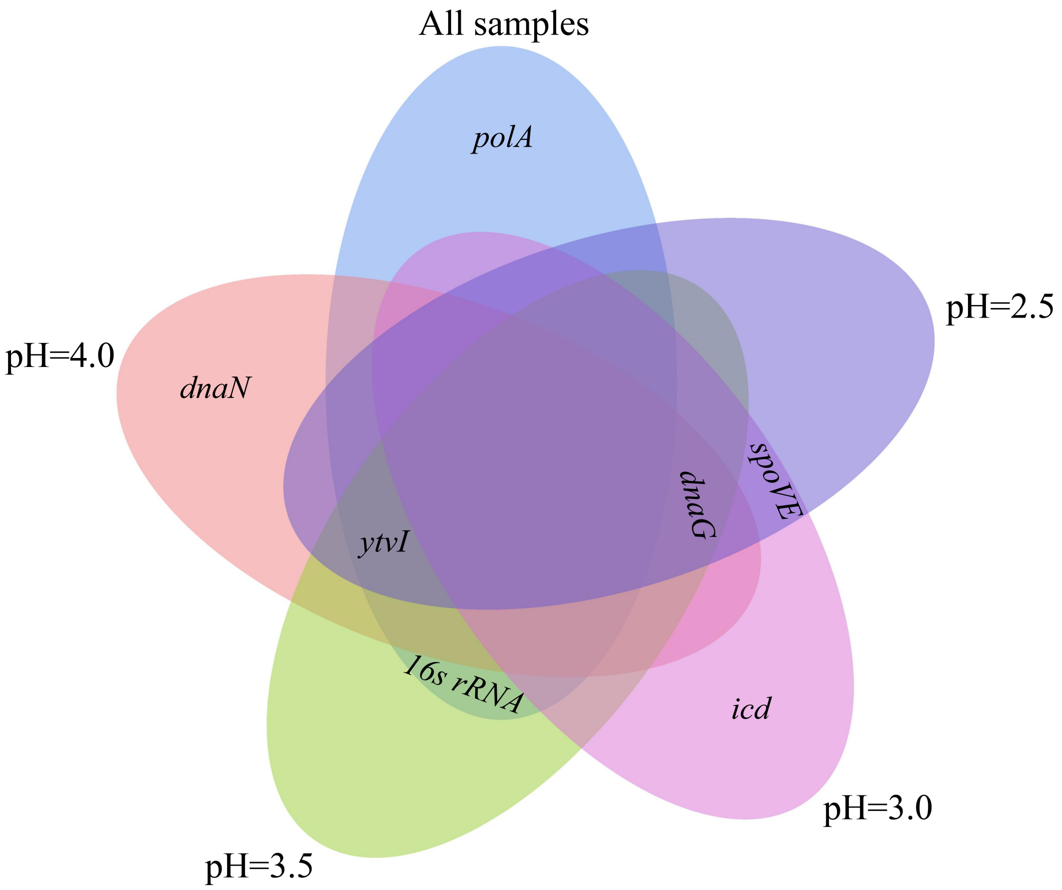

Figure S1. Venn diagram showing the overlap of the three most stable genes in pH 4.0, 3.5, 3.0, and 2.5 stressed *A. acidoterrestris* cells and all samples. The three most stable genes were selected by the overall rankings calculated using the RefFinder method.

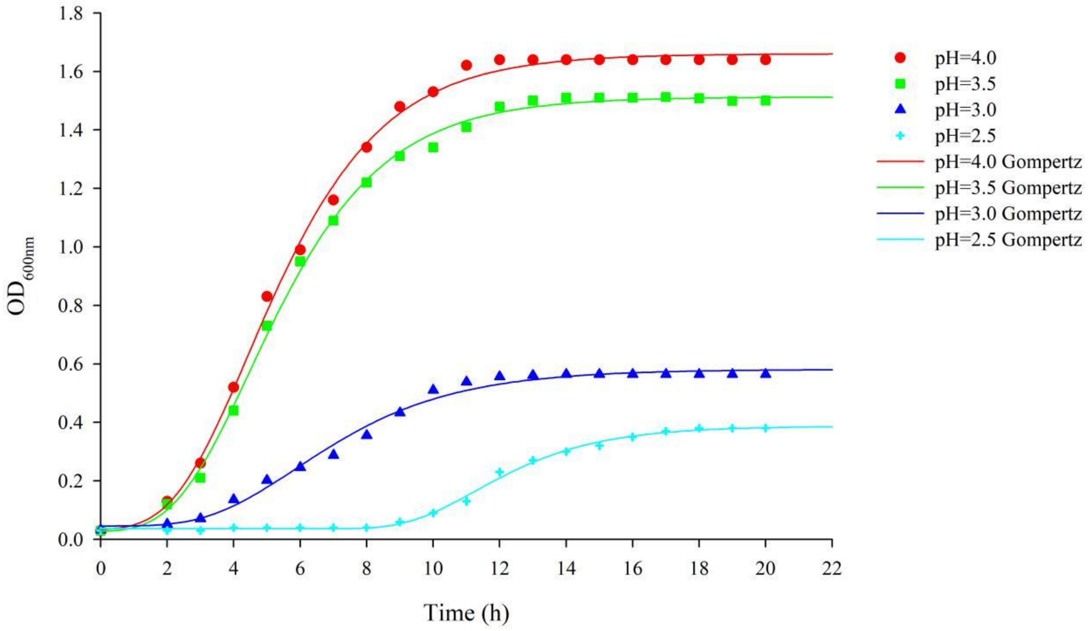

Figure S2. Effects of different pH values on the growth profiles of *A. acidoterrestris*. The symbols represent the means obtained in triplicate from three independent experiments, and the lines represent fitting results of the Gompertz model.
